# How do adult women’s cooking and food preparation skills shape nutrition literacy? A cross-sectional study in Eskişehir, Turkey

**DOI:** 10.1017/S136898002510164X

**Published:** 2025-12-26

**Authors:** Büşra Akbulut, Funda Pınar Çakıroğlu, Eren Canbolat

**Affiliations:** 1 Department of Nutrition and Dietetics, Institute of Health Sciences, Ankara University, Ankara, Turkey; 2 Department of Nutrition and Dietetics, Faculty of Health Sciences, Ankara University, Ankara, Turkey

**Keywords:** Cooking skills, Food preparation, Healthy eating, Nutrition literacy, Public health

## Abstract

**Objective:**

This study aimed to evaluate adult women’s cooking (CS) and food preparation skills (FPS) and their nutrition literacy (NL) levels and to examine the relationship between these two concepts.

**Design:**

Data were collected via face-to-face interviews using a personal information form, the Cooking and Food Preparation Skills scale, and the Evaluation Instrument of Nutrition Literacy on Adults. Data were analysed with SPSS, with *P* < 0·05 considered significant.

**Setting:**

Female adults aged 20–64 years who participated in family support courses in Tepebaşı, Eskişehir, Turkey.

**Participants:**

The study sample consisted of 330 female individuals between the ages of 20 and 64 years who agreed to participate in the survey.

**Results:**

NL was generally adequate (91·8 %), though gaps were identified in portion knowledge (54·2 %) and food label reading (44·2 %, borderline). Higher literacy levels were associated with being younger, more educated and employed (*P* < 0·05). CS and FPS were higher among married women and those with children. Cooking frequency and enjoyment significantly influenced these skills (*P* < 0·05). Those who cooked more often had higher scores in food label reading and basic math (*P* < 0·001) and higher total scores (*P* = 0·049). Participants who enjoyed cooking had better reading comprehension (*P* = 0·030). A weak but significant correlation was found between FPS and general nutrition knowledge, but no strong relationship was observed between overall cooking skills (CS) and total NL.

**Conclusion:**

Although no strong link was found between NL and CS, these skills appear to support healthier eating behaviours. Promoting cooking and food preparation through nutrition education may help improve public health.

Technological development, rising income, population growth, urbanisation, expansion of mass food services and increased female workforce participation have gradually changed individuals’ eating habits^(1)^. Busy schedules, daily stress, reliance on ready-to-eat foods and limited cooking knowledge have reduced home cooking skills (CS) and food preparation skills (FPS)^(2)^. Studies in the USA observed a significant decrease between the 1960s and 2000s in both the time spent preparing meals at home and the percentage of daily energy derived from home-prepared foods^(3)^. Similarly, recent studies in Turkey report an increasing tendency to consume packaged and processed foods, such as ready meals and frozen products, along with more frequent eating outside the home^(1,4)^.

The increasing frequency of eating outside the home raises significant public health concerns because these foods tend to contain higher amounts of energy, total fat, saturated fat and cholesterol. Studies show that improving CS increases the consumption of whole grains, fresh vegetables and fruits^(5)^. Moreover, depending on the cooking method and ingredients, home cooking reduces the consumption of processed foods, which strongly contribute to adverse diet-related health outcomes^(6)^. Analyses of the US NHANES 2007–2010 data indicate that consuming home-prepared meals lowers daily energy intake and reduces sugar and fat consumption^(7)^. Studies show that individuals with better CS consume more fresh vegetables and fewer convenience foods^(8)^. Research indicates that individuals with better CS who cook frequently at home follow healthier eating patterns^(7,9)^.

Nutrition literacy (NL) reflects how individuals obtain, process and understand basic nutrition information and services to make appropriate nutrition decisions^(10)^. Individuals need not only knowledge of nutrition principles but also the skills to comprehend, analyse and apply nutrition information in daily life. Thus, understanding foods and their relationship to health forms the basis of NL^(11)^. Adequate NL positively influences individuals’ food choices, eating habits, diet quality and food preparation practices^(12)^. Higher NL levels promote increased consumption of fruits and vegetables, greater preference for and purchase of healthier foods, and reduced intake of processed and packaged convenience products^(13)^. NL also enables individuals to evaluate information about food choices, interpret food labels, apply food safety measures, use healthy cooking methods and implement nutrition recommendations^(14)^.

Individuals with greater NL and higher FPS tend to adopt healthy eating habits. Adequate NL allows them to access accurate food information and apply it in practice, thereby supporting the development of CS and FPS^(15)^. Including cooking programmes and NL education in school curricula helps children develop healthy eating habits and recognise foods better^(16)^. Since both knowledge and skills shape food choices, examining their relationship is important for understanding healthy eating patterns. The development of these skills is also important for public health. Based on these data, this study hypothesises that higher CS and FPS are positively associated with higher NL levels among adult women living in Eskişehir, Turkey.

## Methods

### Subjects: design, source population and sample

We conducted this cross-sectional study with adult women aged 20–64 years who attended family support courses at the Tepebaşı Social Assistance and Solidarity Foundation in Eskişehir, Turkey, between January 2024 and May 2024. *We calculated the sample size using G* ^*^Power as a reference (effect size 0·2, 95 % power, 5 % error). Because this formula mainly applies to random samples and we used convenience sampling, we treated the calculation as a guideline rather than a strict criterion. We determined the final sample size based on voluntary participation. We included women aged 20–64 years who volunteered to participate, had at least primary school education, had no mental or physical disabilities affecting communication and were responsible for cooking. We required participants to have at least primary school education to ensure they could understand the survey. We used a convenience sampling method based on predetermined inclusion criteria. Participants were informed about the study by course instructors, and those who volunteered were included after giving verbal consent. A total of 339 individuals agreed to participate in the study. We excluded nine participants due to being over 65 years of age (*n* 4) or having incomplete survey data (*n* 5), resulting in a final analytical sample of 330 women.

## Variables/Measurements

### Personal information form

This form was designed to determine adult women’s demographic characteristics (age, education level, marital status, socio-economic status, employment status and whether they have children), dietary habits (number of meals, meal skipping and frequency of eating out) and cooking-related information (frequency of home cooking, who they learned cooking from, how long they spend cooking and whether they enjoy cooking). It consists of a total of twenty-eight questions.

### Cooking and Food Preparation Skills scale

The Cooking and Food Preparation Skills (CFPS) scale, originally developed by Lavelle *et al.* and adapted into Turkish by Keleş and Ok^(4,17)^, was used to assess participants’ skills. The scale consists of two subdimensions: CS (fourteen items; score range 0–98) and FPS (nineteen items; score range 0–133), with a total of thirty-three items. Each item is rated on an eight-point Likert scale ranging from 1 (very poor) to 7 (very good), plus an additional option for ‘never/rarely’. Thus, total scores range from 0 to 231 points, with higher scores reflecting greater CS and FPS. In the Turkish validation study, researchers found an overall Cronbach’s *α* of 0·954, with reliability coefficients of 0·90 for CS and 0·92 for FPS. Confirmatory factor analysis also indicated good model fit (AGFI = 0·93, RMR = 0·042, RMSEA = 0·073).^(4)^ In this study, CFPS showed high internal consistency (*α* = 0·939 total; *α* = 0·858 CS; *α* = 0·929 FPS).

### Evaluation Instrument of Nutrition Literacy on Adults

Cesur and colleagues developed the Evaluation Instrument of Nutrition Literacy on Adults (EINLA) in 2015. It consists of five subsections with a total of thirty-five questions. Each correct answer is scored as one point, while unanswered or incorrect answers receive zero points. NL levels are classified based on total scores as follows: 0–11 points indicate inadequate literacy, 12–23 points borderline and 24–35 points adequate literacy. When evaluating scores for subsections, the general nutrition knowledge and food groups sections are rated as 0–3 points inadequate, 4–7 borderline and 8–10 adequate; the reading comprehension and interpretation section and the food label reading and numerical literacy section are scored 0–2 points inadequate, 3–4 borderline and 5–6 adequate; and the portion sizes section is rated 0–1 point inadequate, 2 borderline and 3 adequate. The tool is applicable to adults aged 18–64 years who have at least a primary school education, and scores range from 0 to 35 points. The scale’s Cronbach’s *α* reliability coefficient is 0·75, item difficulty is 0·55, item discrimination power is 0·73 and the test–retest correlation coefficient is 0·85^(18)^. In the present study, the EINLA demonstrated acceptable internal consistency, with a Cronbach’s *α* of 0·637.

### Data collection

Data were collected on the day of registration through face-to-face and paper-based interviews with participants attending the courses; responses were kept anonymous, interviews lasted 10–15 min and no fee was paid.

### Statistical analysis

We analysed the data using the Statistical Package for Social Sciences (SPSS) version 24.0. We presented qualitative variables as numbers (*n*) and percentages (%), and quantitative variables as mean (X), sd, median, minimum, maximum and interquartile range (Q1–Q3). We applied the Kolmogorov–Smirnov test to assess normality. Since the data were not normally distributed, we used non-parametric tests. We used the Mann–Whitney *U* test to compare two groups and the Kruskal–Wallis test for comparisons involving more than two independent groups. We applied Dunn’s test with Bonferroni correction for multiple comparisons when the Kruskal–Wallis test showed significant differences. We used the Spearman correlation test to analyse the relationship between two quantitative variables. The correlation coefficient (*r*) ranges from –1 to +1, with *r* = 0·00–0·10 considered negligible, *r* = 0·10–0·39 weak, *r* = 0·40–0·69 moderate, *r* = 0·70–0·89 strong and *r* = 0·90–1·00 indicating a perfect relationship. For all statistical tests, a CI of 95·0 % (*P* < 0·05) was accepted.

## Results

In total, 330 adult women completed the study. The participants had a mean age of 41·37 ± 10·9 years, and they were grouped as < 42 and ≥ 42 years accordingly. In total, 51·9 % had a university degree, 33 % had completed high school and 66·1 % were employed. Additionally, 72·1 % of the women had children; among them, 61·8 % had two children, 30·3 % had one child and 7·1 % had three children. The adult women scored an average of 28·1 ± 3·1 (min: 18, max: 35) on the EINLA, indicating scores close to the upper limit. Based on the total EINLA scores, 91·8 % of the participants had an adequate level of NL. Adequate levels were observed in 88·5 % for general nutrition knowledge, 90·3 % for reading comprehension and 90·6 % for knowledge of food groups. However, 54·2 % showed inadequate literacy in portion sizes, and 44·2 % had borderline literacy in the food label reading and basic numeracy subdimension (Table 1).

**Table 1.** General characteristics and nutrition literacy of participants (*n* 330)

Variables		*n*	%
Age groups (years)	< 42 age	156·0	47·3
	≥ 42 age	174·0	52·7
Marital status	Single	88·0	26·6
	Married	242·0	73·4
Educational status	Primary school graduate	50·0	15·1
	High school graduate	109·0	33·0
	University graduate	171·0	51·9
Working status	Working	218·0	66·1
	Not working	112·0	33·9
Having children	Yes	238·0	72·1
	No	92·0	27·9
Number of children (*n* 238)	1	72·0	30·3
	2	147·0	61·8
	3	17·0	7·1
	4	1·0	0·4
	≥ 5	1·0	0·4

		Insufficient		Borderline		Sufficient	
		*n*	%	*n*	%	*n*	%
EINLA assessment	General nutrition knowledge	–		38·0	11·5	292·0	88·5
	Reading comprehension	2·0	0·6	30·0	9·1	298·0	90·3
	Food groups	2·0	0·6	29·0	8·8	299·0	90·6
	Portion sizes	179·0	54·2	92·0	27·9	59·0	17·9
	Food label reading and basic numeracy	110·0	33·3	146·0	44·2	74·0	22·5
	Total score	–		27·0	8·2	303·0	91·8

EINLA, Evaluation Instrument of Nutrition Literacy on Adults.

Among the adult women, 73·3 % reported cooking at home at least 5–7 d a week, and 74·2 % stated that they enjoyed cooking. The participants obtained an average total score of 173·9 ± 41·4 on the CFPS scale. Their average scores were 74·9 ± 17·5 in the CS subdimension and 99 ± 27·7 in the FPS subdimension (Table 2).

**Table 2.** Participants’ findings on cooking and food preparation skills (*n* 330)

Variables		*n*		%
Frequency of cooking at home	5–7 d a week	242·0		73·3
	3–4 d a week	78·0		23·6
	1–2 d a week	10·0		3·1
Enjoyment of cooking	Yes	245·0		74·2
	No	85·0		25·8

CS-FPS, cooking and food preparation skills.

Married participants scored significantly higher in CS (*P* = 0·003), FPS (*P* = 0·004) and total CFPS (*P* = 0·001) compared to single participants. Although age-related differences were not statistically significant, the ≥ 42 age group showed higher average scores. Education level did not significantly affect CFPS or total scores (*P* > 0·05). Participants with children scored significantly higher in CS (*P* < 0·001), FPS (*P* < 0·001) and total CFPS (*P* < 0·001) compared to those without children. Employment status did not significantly affect CFPS scores. As cooking frequency increased, skill levels also rose. Participants who cooked 5–7 d a week scored significantly higher in CS (*P* < 0·001), FPS (*P* < 0·001) and total CFPS (*P* < 0·001) compared to other groups. Similarly, those who enjoyed cooking had significantly higher CS and FPS scores (*P* < 0·001) (Table 3).

**Table 3.** Assessment of participants’ cooking and food preparation skills

CS-FPS	Cooking skills		Food preparation skills		Total score	
	Median	Q1–Q3	Median	Q1–Q3	Median	Q1–Q3
Variables						
Marital status						
Married	81·0	66·75–88·3	107·0	91·0–119·0	187·0	162·75–205·0
Single	72·0	62·0–83·0	96·0	78·0–114·0	170·0	140·25–190·8
P*	**0·003**		**0·004**		**0·001**	
Age groups (years)						
< 42 age	76·0	63·0–86·0	103·0	81·0–117·0	176·5	144·0–202·0
≥ 42 age	81·0	66·0–88·3	106·5	91·0–118·3	183·5	162·8–202·3
P*	0·070		0·218		0·091	
Educational status						
Primary school graduate	76·5	64·75–89·25	101·0	80·0–115·25	177·0	149·75–199·0
High school graduate	81·0	65·0–89·0	108·0	84·0–123·0	189·0	154·5–206·0
University graduate	77·0	64·0–86·0	104·0	88·0–117·0	178·0	152·0–200·0
* P* ^†^	0·297		0·305		0·315	
Having children						
Yes	82·0	67·75–88·3	108·0	91·0–120·0	187·0	164·8–205·0
No	70·5	59·0–81·0	94·5	78·0–113·5	168·0	136·5–189·5
P*	**< 0·001**		**< 0·001**		**< 0·001**	
Working status						
Not working	80·0	64·0–87·0	107·0	87·5–120·5	182·0	150·5–202·0
Working	79·0	65·0–87·0	104·0	86·0–117·0	180·0	153·0–202·0
P*	0·939		0·530		0·765	
Frequency of cooking						
5–7 d a week	82·0^a^	67·0–89·0	108·0^a^	93·0–120·0	188·0^a^	168·8–205·0
3–4 d a week	71·5^b^	58·8–80·3	92·0^b^	75·5–111·3	160·5^b^	134·5–186·5
1–2 d a week	63·5^b^	51·3–72·5	72·0^b^	50·5–105·8	130·0^b^	101·8–172·5
* P* ^†^	**< 0·001**		**< 0·001**		**< 0·001**	
Enjoyment of cooking						
Yes	82·0	68·0–89·0	108·0	93·0–120·0	188·0	168·5–205·5
No	70·0	59·0–79·0	90·0	75·0–110·0	160·0	135·0–181·0
P*	**< 0·001**		**< 0·001**		**< 0·001**	

CS-FPS, cooking and food preparation skills.

Boldface indicates statistically significant results (*P* < 0.05).

^a,b^There is a significant difference between groups containing different letters.

*Mann–Whitney *U* test.

^†^Kruskal–Wallis test.

The results showed that NL levels varied by sociodemographic characteristics. Single participants scored significantly higher than married ones in food label reading and basic numeracy (*P* = 0·002). Participants under 42 years of age performed better in reading comprehension (*P* = 0·043), portion size knowledge (*P* = 0·018) and food label reading (*P* < 0·001), resulting in higher overall NL scores (*P* = 0·003). University graduates achieved significantly higher scores in knowledge of food groups (*P* < 0·001) and food label reading (*P* < 0·001) than other education levels. Participants without children scored significantly higher in reading comprehension (*P* = 0·005), food label reading (*P* < 0·001) and overall NL (*P* = 0·006). Working individuals outperformed non-working individuals in general nutrition knowledge (*P* = 0·009) and total NL score (*P* = 0·010). Women who cooked 5–7 d a week achieved higher scores in food label reading and basic numeracy (*P* < 0·001) and in overall NL (*P* = 0·049). Those who reported enjoying cooking also scored higher in reading comprehension (*P* = 0·030) (Table 4).

**Table 4.** Evaluation of participants’ nutrition literacy levels

EINLA	General nutrition knowledge		Reading comprehension		Food groups		Portion sizes		Food label reading and basic numeracy		Total score	
	Median	Q1–Q3	Median	Q1–Q3	Median	Q1–Q3	Median	Q1–Q3	Median	Q1–Q3	Median	Q1–Q3
Variables												
Marital status												
Married	9·0	8·0–10·0	6·0	5·0–6·0	9·0	8·0–10·0	1·0	1·0–2·0	3·0	2·0–4·0	28·0	26·0–30·0
Single	9·0	8·0–10·0	6·0	5·0–6·0	9·0	9·0–10·0	1·5	1·0–2·75	4·0	3·0–5·0	29·0	27·0–31·0
P*	0·102		0·244		0·632		0·128		**0·002**		0·065	
Age groups												
< 42 age	9·0	8·0–10·0	6·0	5·0–6·0	9·0	9·0–10·0	2·0	1·0–2·0	3·0	3·0–5·0	29·0	27·0–31·0
≥ 42 age	9·0	8·0–10·0	6·0	5·0–6·0	9·0	8·0–10·0	1·0	1·0–2·0	3·0	2·0–4·0	28·0	26·0–30·0
P*	0·794		**0·043**		0·223		**0·018**		**< 0·001**		**0·003**	
Educational status												
Primary school graduate	9·0	8·0–10·0	6·0	5·0–6·0	9·0^a^	8·0–9·0	1·0	1·0–2·0	3·0^a^	1·0–3·25	27·0^a^	24·0–29·0
High school graduate	9·0	8·0–10·0	6·0	5·0–6·0	9·0^a^	8·0–10·0	1·0	1·0–2·0	3·0^a^	2·0–4·0	28·0^b^	26·0–30·0
University graduate	9·0	9·0–10·0	6·0	5·0–6·0	9·0^b^	9·0–10·0	1·0	1·0–2·0	3·0^b^	2·0–5·0	29·0^c^	27·0–31·0
* P* ^†^	0·514		0·457		**< 0·001**		0·541		**< 0·001**		**< 0·001**	
Having children												
Yes	9·0	8·0–10·0	6·0	5·0–6·0	9·0	8·0–10·0	1·0	1·0–2·0	3·0	2·0–4·0	28·0	26·0–30·0
No	9·0	8·25–10·0	5·0	5·0–6·0	9·0	9·0–10·0	1·0	1·0–2·0	4·0	3·0–5·0	29·0	27·0–31·0
P*	0·755		**0·005**		0·591		0·534		**< 0·001**		**0·006**	
Working status												
Not working	9·0	8·0–10·0	6·0	5·0–6·0	9·0	8·0–10·0	1·0	1·0–2·0	3·0	2·0–4·0	28·0	26·0–30·0
Working	9·0	9·0–10·0	6·0	5·0–6·0	9·0	9·0–10·0	1·0	1·0–2·0	3·0	2·0–5·0	29·0	27·0–31·0
P*	**0·009**		0·891		0·071		0·712		0·051		**0·010**	
Frequency of cooking												
5–7 d a week	9·0	8·0–10·0	6·0	5·0–6·0	9·0	8·0–10·0	1·0	1·0–2·0	3·0^a^	2·0–4·0	28·0^a^	26·0–30·0
3–4 d a week	9·0	8·0–10·0	6·0	5·0–6·0	9·0	9·0–10·0	1·0	1·0–3·0	4·0^b^	3·0–5·0	29·0^b^	27·0–31·0
1–2 d a week	9·0	8·0–10·0	5·5	5·0–6·0	9·0	8·0–9·25	1·0	1·0–3·0	4·0^b^	3·0–5·0	29·0^ab^	27·5–30·5
* P* ^†^	0·418		0·371		0·341		0·490		**< 0·001**		**0·049**	
Enjoyment of cooking												
Yes	9·0	8·0–10·0	6·0	5·0–6·0	9·0	9·0–10·0	1·0	1·0–2·0	3·0	2·0–4·0	29·0	26·0–31·0
No	9·0	8·0–10·0	6·0	5·0–6·0	9·0	9·0–10·0	1·0	1·0–2·0	3·0	2·0–5·0	29·0	26·0–30·0
P*	0·421		**0·030**		0·327		0·475		0·487		0·677	

EINLA, Evaluation Instrument of Nutrition Literacy on Adults.

Boldface indicates statistically significant results (*P* < 0.05).

^a–c^There is a significant difference between groups containing different letters.

*Mann–Whitney *U* test.

^†^Kruskal–Wallis test.

Finally, this study examined the relationships between CFPS and the subcomponents of NL. The analysis identified only a positive but weak significant correlation between FPS and general nutrition knowledge (*r* = 0·109, *P* < 0·05). Apart from this, although significant positive correlations were observed among the subdimensions within each scale, the study did not reveal a clear relationship between overall CFPS and NL (Table 5).

**Table 5.** The relationship between cooking and food preparation skills and nutrition literacy

Variables	1.	2.	3.	4.	5.	6.	7.	8.	9.
1. Cooking skills	–	**0·660** ^**^	**0·863** ^**^	0·017	0·079	0·013	−0·041	−0·073	−0·042
2. Food preparation skills		–	**0·940** ^**^	**0·109** ^*^	0·065	0·065	−0·004	−0·101	0·005
3. CS and FPS total score			–	0·081	0·081	0·057	−0·026	−0·097	−0·012
4. General nutrition knowledge				–	**0·154** ^**^	0·038	−0·035	**0·206** ^**^	**0·503** ^**^
5. Reading comprehension					–	0·105	0·035	**0·146** ^**^	**0·393** ^**^
6. Food groups						–	0·017	**0·121** ^*^	**0·452** ^**^
7. Portion sizes							–	0·078	**0·304** ^**^
8. Food label reading and basic numeracy								–	**0·764** ^**^
9. EINLA total score									–

CS, cooking skills; FPS, food preparation skills; EINLA, Evaluation Instrument of Nutrition Literacy on Adults.

Boldface indicates statistically significant results (*P* < 0.05).

*P < 0·05, **P < 0·001.

## Discussion

This study assessed CS, FPS and NL among adult women. Results indicated generally sufficient NL, with some deficiencies in subdimensions, and CFPS were positively associated with healthy eating behaviours.

### Evaluation of participants’ findings related to cooking and food preparation skills

Despite changes in living conditions today, women still mostly undertake the responsibility of cooking and meal preparation. This responsibility enhances women’s CS, as supported by literature showing that women demonstrate higher CS compared to men^(1,19,20)^. Accordingly, the present study, which included only female participants, examined how individuals’ sociodemographic characteristics, nutritional habits and cooking practices influence their CS and FPS.

The research results revealed that participants with children had significantly higher CS and FPS scores compared to those without children (*P* < 0·001). Similarly, married individuals had higher CS and FPS levels than single individuals (*P* < 0·001). On the other hand, we observed no significant differences in CS and FPS according to participants’ age or education level (*P* > 0·05) (Table 3). Although some studies support these findings, other research indicates that age and education level influence CS and FPS. For example, researchers in Australia reported that older individuals demonstrated higher cooking confidence^(21)^. A study in Ireland reported that older individuals demonstrated more advanced CS, while participants with lower education levels scored lower in both CS and FPS^(20)^. A study with pregnant individuals in Turkey reported no significant differences in CS and FPS scores based on age, education level or number of children^(22)^. This situation reveals that CS and FPS may vary according to certain sociodemographic characteristics, but this relationship is not consistent or clear. These inconsistencies are likely influenced by cultural and socio-economic conditions. For example, in some contexts older individuals may retain traditional cooking knowledge, whereas in others younger groups may benefit more from formal nutrition education. Similarly, higher education may increase access to information without always translating into practical CS, while individuals with lower formal education may develop such skills through daily household responsibilities.

In the study, an increase in the frequency of cooking at home had a positive and significant effect on CS and FPS (Table 3). Similar results appear in the literature. Özdemir and Mankan (2022)^(23)^ reported that as culinary students increased the frequency of cooking at home, their CS and FPS scores also increased. Arslan (2023)^(22)^ found that CS and FPS levels varied significantly according to cooking frequency in a study with pregnant individuals. Similarly, a study in Japan involving older adults showed that individuals with low CS cooked at home less frequently^(24)^. A study in the USA with university students found that higher CS positively influenced the frequency of preparing and eating meals at home^(25)^. These data suggest that there may be a bidirectional relationship between cooking frequency and skill level, and that increased frequency of cooking at home has a positive effect on CS and FPS. The enjoyment of cooking also emerges as a determining factor for CS and FPS. The findings show that participants who enjoy cooking have higher levels of CS and FPS (Table 3). Hartmann *et al.* (2013)^(8)^ revealed that enjoying cooking is an indicator of high CS in both genders. Similarly, Özdemir and Mankan (2022)^(23)^ reported that individuals who enjoy cooking had higher CS and FPS scores. These findings suggest that individuals’ enjoyment of the cooking process may be an important motivational factor in enhancing skill levels.

The participants achieved a high total score (173·9 ± 41·4) on the CFPS scale, indicating that they are quite competent in these skills. The subscale scores of CS (74·9 ± 17·5) and FPS (99 ± 27·7) support this conclusion. These results closely align with the CS (78·8 ± 12·6) and FPS (106·7 ± 17·0) scores that Özdemir and Mankan (2022) reported in their study^(23)^. Similarly, Arslan (2023)^(22)^ reported a total scale score of 177·2 ± 31·3 in a study with pregnant individuals. This demonstrates that the current findings are consistent both across different groups and when compared through similar scales. However, Koç (2024)^(26)^ reported lower CS (60·5 ± 20·1) and FPS (75·67 ± 24·35) scores in a study with mothers of children diagnosed with autism spectrum disorder. This suggests that specific life circumstances may influence individuals’ kitchen-related skills. The Turkish validity and reliability study of the scale^(19)^ reported an average total score of 153·7 ± 48·4 for adults, which also supports the high scores observed in the present study. In a comparative international study, researchers found that the CS and FPS scores of students, adults and dietitians in Switzerland differed. Dietitians achieved the highest scores, suggesting that domain knowledge and professional experience may enhance CS and FPS levels^(27)^. The inclusion of only female participants in the current study may have contributed to the high scale scores. The literature reports that gender influences CS and FPS; therefore, the high scores in the present study may reflect women’s greater experience in the kitchen. Beyond individual experience, however, it should be recognised that cooking responsibilities in Turkey, as in many societies, are strongly gendered and predominantly assigned to women. While this division of labour contributes to skill development, it also reflects broader gender inequalities in household responsibilities^(28)^. This gendered context is essential for interpreting the present findings and may limit direct comparability to populations where cooking responsibilities are more equally shared.

### Evaluation of participants’ findings related to Evaluation Instrument of Nutrition Literacy on Adults

Researchers define NL as individuals’ ability to understand the relationship between nutrients and the body, possess knowledge and skills related to healthy and balanced eating, and apply this knowledge in their daily lives. Numerous factors influence the level of NL. Studies conducted across Turkey have reported inconsistent findings on the impact of gender on NL. For instance, while a study found no significant difference between genders^(29)^, another study from Sivas reported that women had significantly higher NL levels than men. Indeed, several other studies also show that women tend to have higher NL levels compared to their male counterparts^(30–32)^. This association may be explained by women’s traditional role in managing family nutrition, their greater emphasis on healthy eating and their heightened awareness of food choices^(33)^.

This study showed that 91·8 % of the female participants had an adequate level of NL. Participants achieved an average total EINLA score of 28·1 ± 3·1. However, the analysis revealed that participants particularly lacked sufficient knowledge about portion sizes, numerical literacy and food label reading (Table 1). This finding aligns with a study from Eskişehir, which reported that although overall NL levels were generally adequate, participants showed the lowest success rates in the subdimensions of portion size (35·4 %) and label reading and basic mathematics skills (23·2 %)^(34)^. Similarly, a study from Aksaray reported that 76·5 % of participants had an adequate level of NL, with the highest scores in the ‘reading comprehension’ subdimension and the lowest in the portion size subdimension^(35)^. A study involving university students also reported similar results, indicating that 82·5 % of participants had an adequate level of NL^(36)^. The differences observed between studies may be due to the participants’ sociodemographic characteristics such as age, gender and education level, their level of knowledge about nutrition, the development level of the regions where the studies were conducted and the measurement tools used. According to the 2018 Turkey Demographic and Health Survey, 41 % of women aged 15–49 years have completed at least high school, with higher rates in the western, northern and central regions. The majority of participants in this study are university graduates, indicating a higher educational level than the national average. Eskişehir, the province where we conducted this study, ranks among the more developed regions in Turkey^(37)^, which likely increased the NL levels of the participants.

When we examined the study results, we found that as the level of education increased, NL levels also increased significantly (*P* < 0·001) (Table 4). Other studies in the literature support this finding as well. For instance, a study involving healthcare professionals reported that NL levels increased as educational attainment reached the postgraduate level^(38)^. As the level of education rises, individuals’ capacity to acquire knowledge about nutrition and their ability to apply that knowledge also increase^(18,32)^. The findings indicate that women’s level of education and marital status positively influence their NL levels.

This study found that marital status did not significantly affect NL (Table 4). However, the literature includes contradictory results on this issue. For instance, a study from Sivas reported that married individuals had higher NL levels than their single counterparts^(38)^. On the other hand, another study reported that single individuals had higher NL levels than married individuals^(39)^. These differing findings suggest that marital status does not have a homogeneous effect on NL and that it may vary depending on individuals’ social, cultural and environmental conditions.

Another key finding of the study shows that age negatively correlates with NL levels (Table 4). Several studies in the literature support this result. Cinemre (2021)^(40)^ reported that NL scores decreased as age increased; Lassetter *et al.* (2015) and Michou *et al.* (2019)^(31,32)^ also noted a decline in NL levels with advancing age. Studies have stated that older individuals experience difficulties in understanding portion sizes, nutrient content and label information, which leads to lower NL levels^(41)^. A study examining the relationship between label reading habits and age found that older adults are less capable of accurately evaluating portion sizes^(42)^. On the other hand, some studies found no significant relationship between age and NL^(43)^. These differences may be due to the fact that NL depends not only on cognitive capacity but also on access to information, literacy level and technology use. Younger individuals’ more effective use of technology and greater access to digital information sources may contribute to higher NL levels in this group.

This study found that employed individuals had significantly higher NL levels than unemployed individuals (*P* = 0·010) (Table 4). Numerous studies in the literature support this result. Döngel (2024)^(34)^ compared NL scores across occupational groups and reported significant differences among civil servants, private sector employees and workers. Michou *et al.* (2019)^(32)^ conducted a study in Greece and found that individuals not employed in any job had lower NL levels compared to those who were employed. These results suggest that engagement in working life positively influences NL by enhancing access to information, fostering a sense of responsibility and promoting interaction with social environments that support health literacy.

### Evaluation of the relationship between cooking and food preparation skills and Evaluation Instrument of Nutrition Literacy on Adults

NL encompasses not only the level of knowledge but also an individual’s ability to apply this knowledge in practice. In this context, a strong relationship is observed between CS, FPS and NL. Begley *et al.* (2019)^(44)^ and Utter *et al.* (2018)^(45)^ reported that CS and FPS positively influence individuals’ nutritional behaviours. Arslan (2023)^(22)^ demonstrated that an increase in health literacy positively affects CS and FPS. Similarly, Gökgöz (2022)^(46)^ stated that nutrition knowledge has a positive impact on both FPS and the attitude and behaviour subdimensions of NL. This study examined the relationships between women’s NL levels, portion knowledge and FPS. Although studies evaluating these three variables together are quite limited in the literature, the current findings reveal the multidimensional nature of the field.

The current literature addresses the effect of NL levels on individuals’ eating behaviours from various perspectives. For example, Dönmez (2024)^(47)^ conducted a study and reported that NL scores decreased as the frequency of eating out increased. Similarly, Dilşat *et al.* (2023)^(33)^ found that individuals who frequently eat outside the home have lower NL scores, and that the tendency to make healthy choices increases as NL improves. These findings indicate that individuals’ NL levels are closely related not only to knowledge but also to behavioural tendencies. Begley *et al.* (2019)^(44)^ reported that individuals with low skills in meal planning and management also have weaker CS and are less likely to take responsibility during shopping. Utter *et al.* (2018)^(45)^ reported that CS are associated with healthier eating behaviours in the long term. In this context, an increase in the level of NL can lead to positive changes both in food choices and in home meal preparation behaviours. In the current study, participants’ high overall nutrition knowledge corresponded with their high NL scores, consistent with the literature (Table 5).

Research indicates that components such as nutrition knowledge and FPS positively contribute to individuals’ diet quality. A study in Canada found a positive relationship between food label use and Health Literacy (HL) level^(48)^, whereas Vijaykumar *et al.* (2013)^(49)^ reported an inverse relationship between food label use and HL. A study in Turkey found that individuals who received nutrition education had significantly higher food label reading scores, one of the subdimensions of NL^(50)^. These findings indicate that correctly reading food labels directly relates to healthy food selection. Conversely, a study found that individuals with high NL levels consumed more convenience foods. This result shows that a high level of NL alone does not guarantee healthy choices, and that factors such as living conditions, work life and practicality are also effective in food preferences^(29)^. Similarly, it is as important to transform knowledge into life practice as it is to possess the knowledge itself. Indeed, Cesur *et al.* (2015)^(18)^ emphasise that having sufficient knowledge alone is not enough to develop healthy behaviour. However, this study found no statistically significant relationship between NL level and the total scores of CFPS (Table 5). This finding suggests that the relationship between these variables is not direct and linear but rather complex and multidimensional. In addition, behavioural variables such as motivation, enjoyment of cooking or time constraints, as well as environmental factors such as socio-economic status and access to healthy foods, may mediate the relationship between NL and CFPS. This weak association may be explained by several factors. In the Turkish cultural context, CS and FPS are often acquired informally through family traditions rather than through formal nutrition education, which may weaken their association with NL. Furthermore, gaps in the education system regarding nutrition and health literacy may limit individuals’ ability to translate cooking experience into nutrition knowledge. In addition, the measurement tools focus on different constructs, with NL assessing knowledge and comprehension, while CFPS assess practical skills. These differences may explain why the correlation observed in our study was relatively low. NL is a comprehensive concept that includes not only the individual’s level of knowledge but also their capacity to understand, apply and transform this knowledge into behaviour. Therefore, individuals with a high level of NL are expected to have not only theoretical knowledge but also practical skills such as portion control, label reading, healthy cooking methods and numeracy^(51)^. As a result, these findings suggest that the relationships between NL, CS and FPS are multi-layered and complex, and that they are influenced by individuals’ knowledge, skills and environmental conditions. Therefore, interventions aimed at increasing NL should focus not only on transferring knowledge but also on developing behavioural skills.

The study has some limitations, including evaluating CS and FPS solely through participants’ self-reported information without practical application and restricting the sample to women living in the province of Eskişehir. The inclusion of only women attending family support courses may limit the generalisability of the findings. Additionally, the use of a non-random, volunteer-based sample and the inclusion criterion of having at least primary school education may further reduce generalisability. The criterion of having at least primary school education may have led to a selection bias due to the exclusion of individuals with lower literacy levels. Therefore, some of the results are likely to be specific to this particular socio-economically defined population and should be interpreted with caution. Nevertheless, several observed patterns (e.g. the influence of education level and cooking frequency) are consistent with findings from broader contexts, suggesting that the study provides both local insights and contributions of wider relevance. Future studies involving different demographic groups will further enrich the literature on the subject. In the current literature, there are not a sufficient number of studies examining the relationship between adult women’s CS and FPS and their NL levels. Therefore, the results of this study make an important contribution to the literature.

### Conclusion

This study examined the levels of CS, FPS and NL among adult women in Eskişehir, Turkey, specifically those attending family support courses. The study included participants who volunteered and had at least a primary education; therefore, the findings reflect the characteristics of this specific population. The findings obtained indicate that CS and FPS are positively associated with individuals’ healthy eating behaviours. The study observed that sociodemographic characteristics, such as regularly cooking at home, enjoying cooking and having children, increase CS and FPS levels. Although the study found that NL generally reached a sufficient level, participants showed deficiencies in certain areas, such as portion control and label reading. While the study found no significant relationship between the total scores of NL and CFPS, the subdimension findings revealed weak but meaningful associations. These results suggest that these skills have multidimensional connections with eating behaviours. As a result, in order to support healthy eating habits, nutrition education should be structured not only based on knowledge but also to include practical CFPS. In line with national strategies such as the ‘Healthy Nutrition and Active Life Program’ of the Turkish Ministry of Health, integrating NL training into community-based education (e.g. family support courses and schools) may further enhance public health outcomes. This approach can contribute to the improvement of public health by enabling individuals to make more conscious and healthy choices from food selection to the preparation process. In addition, the results highlight the need for concrete actions in health policies, educational programmes and gender roles. Policymakers could support interventions that integrate NL and CS training into public health initiatives, while educators may include such content in curricula and adult education. Furthermore, encouraging men as well as women to take responsibility in cooking would help reduce gender inequalities and promote shared responsibility for healthy eating within households.
